# Metabolic reprogramming in colorectal cancer: a review of aerobic glycolysis and its therapeutic implications for targeted treatment strategies

**DOI:** 10.1038/s41420-025-02623-5

**Published:** 2025-07-14

**Authors:** Boran Pang, Hao Wu

**Affiliations:** 1 Center for Difficult and Complicated Abdominal Surgery, Shanghai Tenth People’s Hospital, Tongji University School of Medicine, Shanghai, China; 2 Heilongjiang Clinical Research Center for Breast Cancer, Harbin Medical University Cancer Hospital, Harbin, China

**Keywords:** Cancer immunotherapy, Colon cancer

## Abstract

Colorectal cancer (CRC) remains a significant oncological challenge, being among the foremost contributors to cancer-related mortality worldwide. This review summarizes our current knowledge regarding how metabolic reprogramming, specifically the Warburg effect, contributes to CRC pathobiology and explores its therapeutic relevance. Metabolic reprogramming in CRC is characterized by a shift from oxidative phosphorylation to glycolysis, termed the Warburg effect. Driven by the tumor microenvironment (TME), this adaptation enhances cancer cell proliferation through accelerated ATP generation, biosynthesis support, and redox balance. Key glycolytic enzymes, namely hexokinase, phosphofructokinase, pyruvate kinase, and lactate dehydrogenase are now prioritized as therapeutic targets in CRC treatment strategies. Diagnostic modalities utilizing CRC’s altered metabolism such as 18F-fluorodeoxyglucose positron emission tomography (18F-FDG PET/CT) and metabolomic analysis of circulating metabolites, improved early detection through enhanced sensitivity and specificity. These approaches reveal CRC’s distinct metabolic signatures, enabling precise disease stratification and management. Therapeutic strategies targeting the EMP pathway show preclinical efficacy in overcoming CRC-associated chemoresistance and radioresistance. Modulation of EMP-regulating pathways (AKT, AMPK, mTOR) provides additional therapeutic opportunities. However, CRC’s metabolic heterogeneity demands multi-targeted approaches. The development of targeted therapies must consider the potential off-target effects on normal tissues that rely on EMP, necessitating a careful balance between therapeutic efficacy and safety. In summary, this review underscores the complexity of metabolic reprogramming in CRC and the need for a nuanced approach to target these pathways effectively. Subsequent investigations should prioritize defining tumor-selective metabolic vulnerabilities and engineering multi-pathway interventions that spare normal tissues, ultimately advancing therapeutic precision in CRC management.

## FACTS

- Metabolic reprogramming driven by dysregulated glucose, lipid, and glutamine metabolism fuels CRC pathogenesis through key enzymes, positioning multi-targeted EMP inhibition as a core therapeutic strategy.
- Persistent mitochondrial OXPHOS in CRC cells enables metabolic compensation that undermines EMP-targeted therapies, necessitating combinatorial inhibition of complementary pathways (e.g., TCA cycle, PPP) for durable efficacy.
- On-target toxicities of EMP inhibitors in glycolytic-dependent normal tissues (e.g., brain, retina) mandate tumor-selective strategies and multi-target regimens to optimize therapeutic indices.

## QUESTIONS

- How do dysregulated glucose, lipid, and glutamine metabolism cooperatively drive CRC progression via specific metabolic enzymes? What signaling networks integrate these pathways?
- To what extent does OXPHOS-mediated metabolic compensation limit EMP-targeted monotherapies in CRC? Which compensatory pathways (TCA/PPP/glutamine) are most critical?
- How can tumor-selective targeting of EMP nodes spare glycolytic-dependent normal tissues? What delivery strategies or combination regimens maximize therapeutic indices?

## Introduction

Colorectal cancer (CRC) stands as a formidable malignancy, commanding a significant position among the leading causes of cancer-related mortality worldwide. Recent epidemiological data from the United States (2023) estimate ~1 million annual CRC deaths, positioning it as the second most prevalent cancer in women and third in men [1]. Alarmingly, its incidence continues to rise in adults, underscoring an urgent public health challenge [2].

CRC, being a prevalent tumor of the digestive system, is a prime candidate for research into its metabolic reprogramming, a hallmark of cancer that encompasses alterations in various metabolic pathways such as glycolysis, oxidative phosphorylation (OXPHOS), etc [3]. These adaptions drive tumor proliferation, survival, and migration by fundamentally reshaping cellular biological characteristics [4]. The tumor microenvironment (TME) is recognized as one of the most critical factors in the initiation and progression of malignant tumors, with a profound interplay with energy metabolism. Aerobic glycolysis, a predominant glucose metabolism pathway, serves as the primary means by which cells harness energy. However, the oncogenic transformation of normal cells is frequently accompanied by a profound remodeling of metabolic pathways [5].

In CRC, tumor cells exhibit a hallmark metabolic shift toward the Embden–Meyerhof–Parnas (EMP) pathway for energy production, even under oxygen-replete conditions, a phenomenon termed the Warburg effect [3]. By favoring EMP over OXPHOS, CRC cells prioritize rapid ATP generation, biosynthesis of macromolecular precursors (e.g., nucleotides, lipids), and maintenance of redox homeostasis, enabling adaptation to dynamic microenvironmental stresses. Notably, fructose metabolism synergistically amplifies this metabolic reprogramming by bypassing EMP rate-limiting steps (e.g., phosphofructokinase (PFK)) and enhancing nicotinamide adenine dinucleotide phosphate (NADPH)-mediated redox buffering capacity, thereby exacerbating CRC aggressiveness and positioning ALDOB inhibition as a therapeutic vulnerability [6, 7]. Elucidating the molecular drivers of this metabolic reprogramming is crucial for developing therapies that target CRC’s unique metabolic dependencies. Such reprogramming is inextricably linked to genetic mutations, driven by dysregulated oncogenes and inactivated tumor suppressors, which rewire cellular metabolism to fuel carcinogenesis [8, 9].

Metabolomics, including metabolic imaging, and computational modeling have revolutionized CRC metabolic reprogramming research, enabling novel therapeutic and preventive strategy development. Metabolic-targeted therapies, including pathway modulators, enzyme inhibitors, and metabolic agents, represent a novel therapeutic frontier. This convergence with tumor immunology has amplified exploration of CRC’s therapeutic potential [10]. Recent studies identify self-renewing, proliferative, and differentiation-capable cancer stem cells as critical drivers of tumorigenesis, metastasis, and therapy resistance [11].

This review aims to synthesize the current understanding of the role of metabolic reprogramming in CRC, highlighting its mechanisms and exploring its implications for targeted treatment strategies. Through analyzing tumor-specific metabolic divergence from normal cellular processes, we map actionable intervention pathways for CRC prevention and treatment, while advancing conceptual understanding of its pathobiological complexity.

## Glucose metabolic reprogramming in CRC

Glucose catabolism is a pivotal process for cellular energy production and is primarily conducted through three pathways: glycolysis, OXPHOS, and the pentose phosphate pathway (PPP). These interconnected pathways undergo pathological rewiring during CRC oncogenesis, driving malignant transformation through dysregulated energy flux [12].

The glucose metabolic reprogramming in CRC centers on the EMP-TCA cycle nexus, glucose uptake is facilitated initiated by glucose transporter 1 (GLUT1)-mediated glucose influx [13]. Hexokinase (HK) phosphorylates glucose to glucose-6-phosphate (G6P), which is further metabolized to 2-phosphoglycerate by enolase and then to phosphoenolpyruvate (PEP) [14]. Pyruvate kinase-mediated (PKM) PEP-to-pyruvate conversion reveals dual metabolic fate: pyruvate dehydrogenase (PDH)-dependent acetyl-CoA production for TCA cycle entry versus lactate dehydrogenase (LDH)-driven lactate (LA) generation, epitomizing the Warburg phenotype.

**Fig. 1:**
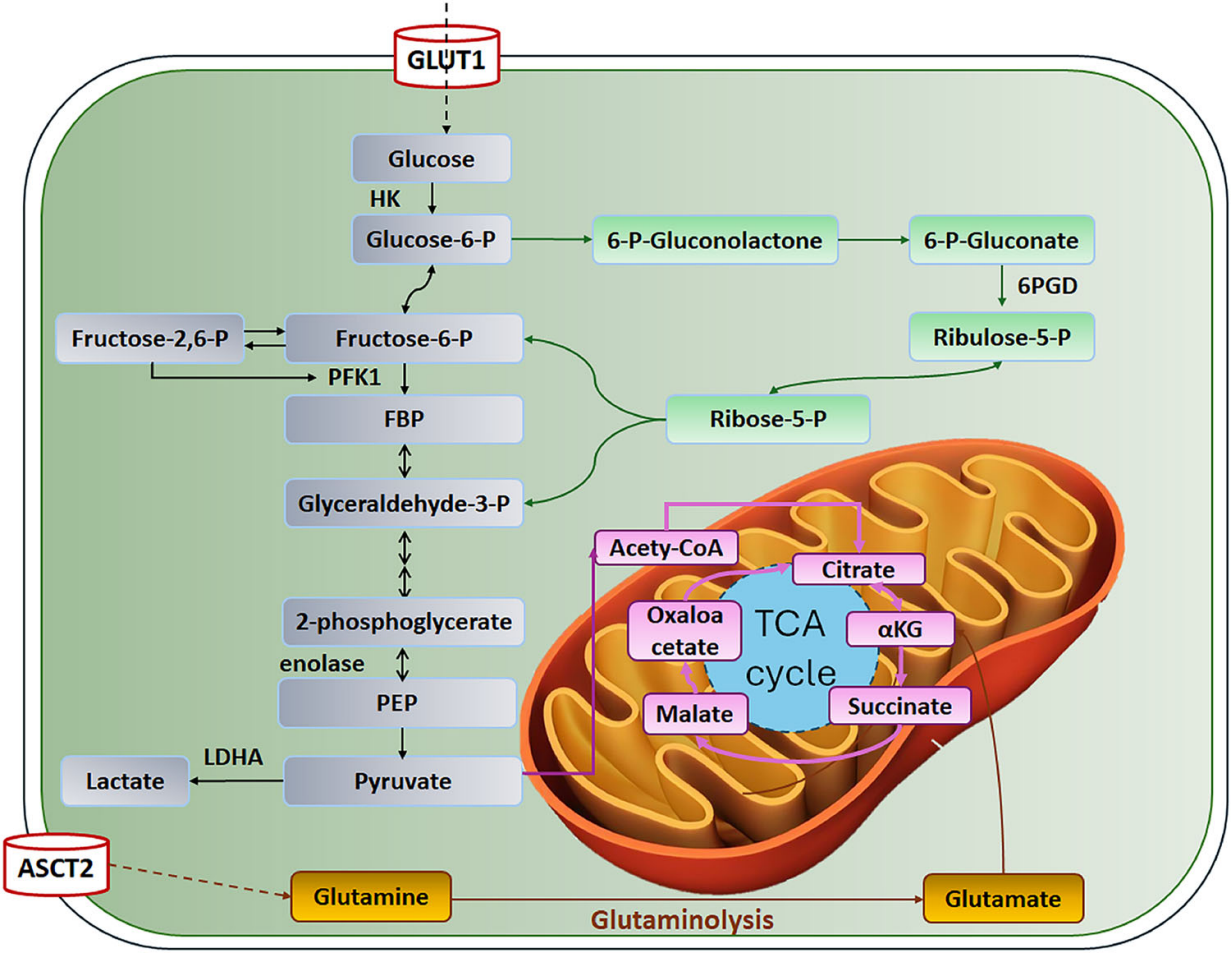
Metabolic reprogramming in CRC: interplay of EMP, PPP, oxidative phosphorylation, and TCA cycle. Glucose enters via GLUT1 and is phosphorylated by HK to G6P, which feeds into PPP (providing nucleotide precursors) or glycolysis. Glycolysis is enhanced by fructose-2,6-bisphosphate activating PFK1. The resulting pyruvate is converted to lactate by LDHA or enters mitochondria for the TCA cycle, generating energy and biosynthesis intermediates. Glutamine imported via ASCT2 undergoes glutaminolysis to replenish TCA cycle metabolites. Solid lines: metabolic processes; Dashed lines: metabolite transport across cellular membranes.

The TCA cycle orchestrates metabolic plasticity through citrate-mediated lipogenesis and α-ketoglutarate (αKG)-derived glutamate synthesis via glutamate dehydrogenase, contributing to amino acid metabolism [15]. The diagram underscores the metabolic plasticity in CRC, where intermediates are rerouted for anabolic purposes, supporting tumor growth and proliferation (Fig. 1).

### The Warburg effect in CRC

The Warburg effect, aerobic glycolysis under normoxic conditions, is a distinctive metabolic trait observed in cancer cells. In CRC cells, prioritized LA-generating glycolytic ATP production persists despite oxygen sufficiency, fueling rapid proliferation and biosynthesis demands [16]. Extensive research has indicated a reduction in glucose oxidation and an enhancement of the Warburg effect and PPP in CRC. Early-stage molecular upregulation of hypoxia-inducible factor-1α (HIF-1α), GLUT1, pyruvate kinase M2 (PKM2), and lactate dehydrogenase A1 (LDHA) observed in premalignant lesions implies that Warburg effect activation precedes malignant transformation [17, 18].

In CRC, overexpression of glucose transporters and key glycolytic enzymes drives the Warburg effect, resulting in accelerated glycolytic flux. The accumulation and diversion of glycolytic intermediates are utilized for the synthesis of cancerous biomass, rapid ATP production, and LA accumulation, which collectively promote tumor progression. TME acidosis stemming from this metabolic shift promotes tumor progression, induces therapeutic resistance, and compromises antitumor immune responses [19].

While glucose competition in the TME drives T cell dysfunction, parallel depletion of amino acids (e.g., glutamine), and fatty acids through transporters compromises immune cell activity. Additionally, the high levels of LA, low pH, hypoxia, and reactive oxygen species (ROS) prevalent in the TME can impair T cells and natural killer cells while polarizing tumor-associated macrophages into a pro-tumoral M2-like phenotype, collectively facilitating immune evasion [20]. These findings collectively highlight therapeutic potential in targeting Warburg effect regulators through pharmacological or epigenetic approaches, with key glycolytic enzymes (HK2, PKM2, LDHA) representing strategic intervention points to disrupt both metabolic fueling and immunosuppressive mechanisms in CRC [21].

Notably, fructose metabolism synergizes with the Warburg effect to amplify CRC aggressiveness. While glucose-driven aerobic glycolysis generates LA via LDHA, fructose bypasses glycolysis rate-limiting steps (e.g., PFK), ensuring sustained ATP and biomass production under nutrient stress [22]. This metabolic crosstalk enhances redox balance through NADPH generation and stabilizes hypoxia-inducible factors, collectively promoting tumor survival, immune evasion, and metastasis. Therapeutic strategies targeting ALDOB or fructose uptake could disrupt these interconnected pathways, offering a promising approach to counteract CRC’s metabolic resilience and improve clinical outcomes [23].

### Oxidative phosphorylation in CRC

Emerging studies delineate metabolic bifurcation in cancer cells, where a subset preferentially engages OXPHOS over glycolysis. This divergence is amplified through intratumoral heterogeneity that sustains coexisting metabolic states. Empirical evidence has established that CRC cells exhibit a greater reliance on OXPHOS versus normal colonocytes, exceeding conventional glycolytic predilection [24]. Crucially, stromal interactomes, particularly cancer-associated fibroblasts (CAFs), are of significant consequence in the facilitation of tumor progression.

The “reverse Warburg effect” delineates bidirectional metabolic crosstalk between cancer cells and stromal cells, wherein cancer cell-derived ROS induce oxidative stress in CAFs. This triggers CAF metabolic reprogramming towards aerobic glycolysis, generating energy-rich metabolites, including pyruvate, LA, fatty acids, and ketone bodies. MicroRNA-1 downregulation coordinates this stromal adaptation, while monocarboxylate transporter upregulation facilitates LA transfer from CAFs to tumor cells. This intercellular metabolic symbiosis suggests a stromal cell-cancer cell metabolic axis that is instrumental in fostering tumorigenesis [25, 26].

The seminal work by Nenkov et al. [27] elucidates the intricate crosstalk between colon cancer cells and stromal cells, underscoring the reverse Warburg effect. Their study delineates how perivascular TME fluctuations drive metabolic remodeling, creating nutrient-oxygen gradients that spatially regulate oxygen availability. Notably, perivascular cancer cells exhibit elevated OXPHOS activity compared to distal counterparts, revealing microenvironment-governed metabolic zonation [28]. These findings underscore the spatial heterogeneity of metabolic activity within tumors and its implications for tumor biology and therapeutic targeting.

These studies emphasize the significance of OXPHOS in CRC, the role of the reverse Warburg effect, and the metabolic heterogeneity within tumors, offering a comprehensive foundation for further investigation into the metabolic underpinnings of cancer progression and therapeutic resistance.

### The multifaceted role of the pentose phosphate pathway in CRC and Its therapeutic potential

The PPP, alternatively termed the phosphogluconate pathway branches from glycolysis at G6P to generate fructose-6-phosphate and glyceraldehyde-3-phosphate [29]. Unlike glycolysis and aerobic glucose oxidation, the PPP does not generate ATP; instead, it produces reduced NADPH for cellular redox balance and ribose-5-phosphate for nucleic acid synthesis, respectively [30].

In the context of CRC, the PPP serves multiple critical roles. NADPH, a product of the PPP, maintains cellular antioxidant defenses by reducing glutathione, protecting cancer cells from oxidative damage. Concurrently, ribose-5-phosphate, another PPP product, provides essential precursors for nucleotide synthesis required for rapid cancer cell proliferation [31, 32].

Recent studies have underscored the critical involvement of the PPP in CRC. Elevated expression of rate-limiting enzyme glucose-6-phosphate dehydrogenase drives PPP activation, promoting tumorigenesis and progression. The pathway sustains genomic stability in aneuploid cells through mitotic NADPH production that prevents chromosomal aberrations while directly fueling CRC proliferation via nucleotide biosynthesis. Additionally, the PPP’s involvement in nucleotide synthesis is directly associated with the proliferation and survival of CRC cells. The transketolase, a non-oxidative PPP component regulating ribose-5-phosphate and NADPH homeostasis, has surfaced as a promising therapeutic target [33].

The PPP’s integration with CRC metabolism is exemplified through cysteine metabolism, a glutathione synthesis prerequisite requiring substantial NADPH from PPP-mediated cystine-to-cysteine conversion. This reliance underscores the metabolic vulnerability of CRC cells, particularly those with high SLC7A11 expression, offering a potential avenue for targeted therapeutic interventions [34, 35].

The PPP’s significance extends to the broader scope of cancer prevention and treatment. NADPH and ribose-5-phosphate, products of the PPP, are vital in modulating the DNA damage response, cellular metabolism, and the proliferation of cancer cells. Consequently, multiple PPP enzymes have been identified as promising therapeutic targets, functioning dually as metabolic catalysts and regulators of diverse cellular processes, positioning them as strategic targets for anticancer drug development [36].

## Diagnostic methods based on metabolic reprogramming in CRC

The metabolic reprogramming in CRC creates diagnostic opportunities by exploiting cancer-specific metabolic alterations. These approaches elucidate tumor biology, enabling stage-specific identification and metabolic profiling. Subsequent sections detail advanced imaging and metabolomic applications that utilize CRC’s distinct metabolic signatures to enhance detection accuracy and therapeutic management.

### Positron emission tomography (PET) with 18F-fluorodeoxyglucose (18F-FDG)

18F-fluorodeoxyglucose positron emission tomography combined with computed tomography (18F-FDG PET/CT) has become a cornerstone in CRC diagnostics, offering insights beyond mere morphological changes. It provides a comprehensive view into the glycolytic metabolism of glucose uptake across various tissue cells, which is crucial for early and accurate diagnosis of malignant tumors. This advanced imaging technique has been shown to outperform traditional CT or MRI in detecting recurrent lesions, with particular clinical utility in CRC surveillance [37]. Integration with serum biomarkers like carcinoembryonic antigen augments its diagnostic precision, making it an indispensable asset in the diagnostic arsenal for CRC. Moreover, PET/CT’s diagnostic value extends to the identification of metastatic lesions, critically informing stage-appropriate treatment stratification [38].

The advent of 18F-labeled glutamine tracers expands diagnostic capabilities for 18F-FDG-negative CRC tumors, addressing previous metabolic imaging limitations. This innovative approach has been validated in both CRC patients and animal models, demonstrating its effectiveness [39, 40]. Concurrently, PET/magnetic resonance imaging (PET/MRI) emerges as a safer and more effective alternative to PET/CT with enhanced soft-tissue resolution and multimodal functional imaging. While primarily oncological tools, both PET/CT and PET/MRI demonstrate expanding utility in cardiovascular and neurological disease diagnostics, broadening their clinical applicability [41, 42].

Future studies must comprehensively evaluate imaging modalities’ clinical potential in CRC, particularly defining optimal applications where PET/CT and PET/MRI demonstrate maximal diagnostic superiority. Critical priorities include establishing integration protocols for routine screening pathways, assessing cost-effectiveness across healthcare systems, and evaluating longitudinal impacts on treatment personalization and survival metrics. These areas of research will not only enhance our understanding of CRC but also guide the development of more targeted and effective diagnostic and treatment strategies [43].

### Circulating metabolite detection

Circulating metabolite detection holds promise as a diagnostic method for CRC. Metabolomic profiling enables the detection of endogenous small-molecule metabolites integral to physiological processes, whose concentration patterns mirror systemic biochemical states. By collecting and analyzing changes in these metabolites, potential biomarkers associated with diseases can be identified, facilitating clinical diagnosis and disease stratification [44].

Comparative plasma metabolite analysis between CRC patients and healthy controls identifies differentially expressed species: leucine-enkephalin, 5-hydroxytryptamine, and propionic acid imidazole that are upregulated, while perfluorooctanesulfonic acid, 2-linoleoylglycerol (18:2), and sphingosine that are downregulated [45]. This indicates that metabolomics-based circulating biomarkers have the potential to serve as early diagnostic indicators for CRC.

## Inhibition of glycolysis pathway for the treatment of CRC

The pronounced glycolysis dependency of CRC cells under normoxic conditions constitutes an exploitable metabolic vulnerability. Targeting the key enzymes of this pathway holds promise for disrupting the energy supply and biosynthetic needs of CRC cells, offering a strategic approach to combat the disease.

### Inhibition of key enzymes in the EMP pathway in CRC

Elevated expression of critical EMP pathway enzymes drives therapeutic resistance in CRC, particularly to radiotherapy and chemotherapy [46]. Emerging evidence demonstrates that pharmacologically inhibiting these enzymes—HK, PFK, and PK, all overexpressed in CRC—effectively suppresses tumor proliferation, positioning them as prime therapeutic targets for EMP-directed CRC treatment.

HK, the first rate-limiting enzyme of the EMP pathway, has four isoforms with distinct distributions, with HKII showing predominant association with malignancy and marked overexpression in CRC [47]. Phosphorylation of HKII’s hydrophobic N-terminus enables outer mitochondrial membrane binding via voltage-dependent anion channel (VDAC) interaction, driving glycolytic flux while competitively inhibiting pro-apoptotic Bax-VDAC binding. This dual mechanism reduces cytochrome C release and mitochondrial membrane potential, conferring apoptosis resistance. Studies have shown that silencing HKII in CRC cell lines such as HT-29, SW480, HCT-15, RKO, and HCT-116 can effectively halt their proliferation and high EMP activity [48]. Clinical evidence links elevated HKII expression to advanced CRC features including tumor volume, invasion depth, staging, and lymph node metastasis, establishing its dual role as both a prognostic marker and therapeutic target.

PFK, the most important rate-limiting enzyme of the EMP pathway, exists as PFK1 and PFK2 isoforms. The PFK2 subtype PFKFB3 shows marked overexpression in malignancies including CRC, with its regulation linked to oncogenic activation (e.g., RAS, CDH1) [49]. Zhao et al. [50] found that interleukin-6 (IL-6) blockade disrupts EMP-mediated CRC progression: the transcription of key EMP genes in CRC cells is upregulated by IL-6 stimulation, promoting proliferation and metastasis; knocking out PFKFB3 can eliminate the above effects of IL-6. Augmented EMP flux in tumors induces concomitant upregulation of PFK1 expression, demonstrating a positive correlation with pathway metabolic efficiency [51]. Although PFK-targeted therapies show growing therapeutic promise, their tissue-specific mechanisms and EMP inhibition modalities require further elucidation.

PK, the EMP’s rate-limiting enzyme, converts PEP to pyruvate through substrate-level phosphorylation, yielding ATP. Among the four PK isoforms (PKL, PKR, PKM1, and PKM2), PKM2 is predominantly present in most tumor cells [52]. Beyond its canonical EMP role, PKM2 exhibits reduced catalytic activity, leading to the accumulation of intermediates and their diversion to the PPP for macromolecule biosynthesis, accelerating the proliferation of tumor cells. PTBP1 knockdown triggers PKM2-to-PKM1 isoform switching and a high proportion of PKM1/PKM2 suppress EMP, inhibiting cell growth, and inducing apoptosis [53]. Ginés et al. [54] demonstrate that PKM2 silencing in HT29, SW480, and HCT116 enhanced oxaliplatin sensitivity proportional to PKM2 suppression levels. These findings establish PKM2 downregulation as a strategic approach to inhibit EMP and restore chemosensitivity in CRC treatment resistance.

LDH comprises three isoforms (A, B, C), with LDHA demonstrating predominant overexpression in CRC tissue [55]. It promptly catalyzes the transfer of LA produced by PA to the outside of the cell, reducing the accumulation of LA and promoting efficient EMP. Qin et al. [13] identified nutmeg extracts as LDHA-targeting inhibitors that suppress EMP-driven ATP production and glucose uptake in CRC cells. In 5-Fluorouracil (5-Fu)-resistant CRC models, LDHA upregulation correlates with chemoresistance, while miR-34a-mediated LDHA inhibition restores 5-Fu sensitivity through EMP suppression [56]. These findings position LDHA inhibition as a strategic approach to overcome treatment resistance via metabolic targeting in CRC therapeutics.

Glucose transporters protein (Gluts) mediate glucose transport across cell membranes along concentration gradients. Among these, Glut1 is most closely related to sugar metabolism and is overexpressed in various solid tumors [57]. HIF-1α pathway inhibition reduces Glut1 transcription in CRC cells, suppressing glucose uptake and inducing apoptosis [13]. Glut1 drives multidrug resistance through dual mechanisms: upregulating drug efflux pumps (P-gp, MRP) to maintain subtherapeutic intracellular drug concentrations and elevating the Bcl-2/Bax ratio to enhance anti-apoptosis capacity. Drug-resistant CRC cells exhibit Glut1 overexpression compared to parental lines, with low-dose 5-Fu inducing dose-dependent Glut1 upregulation to accelerate glucose uptake. Using a specific inhibitor WZB117 to silence Glut1, drug-resistant cells regain sensitivity to 5-Fu [58]. As a potential target for CRC treatment, Glut1 has important clinical value, and its study is expected to provide important molecular biological information for the diagnosis and treatment of CRC.

**Fig. 2:**
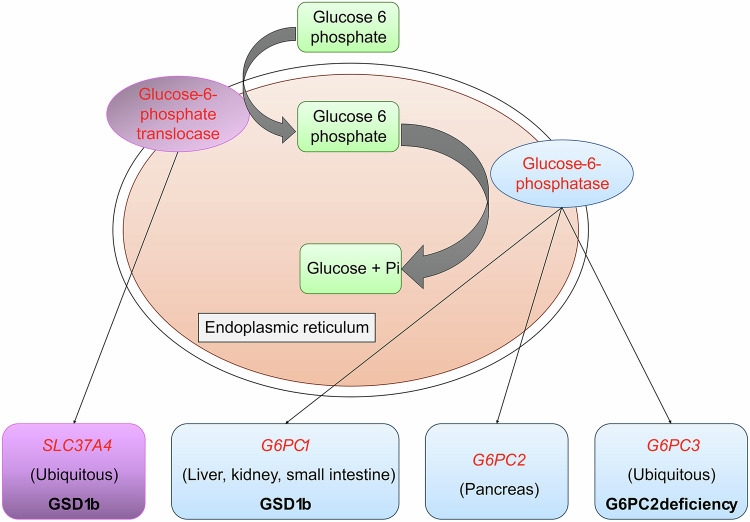
Glucose transport mechanism across the endoplasmic reticulum membrane. Cytosolic glucose-6-phosphate (G6P) enters the ER via glucose-6-phosphate translocase (G6PT/SLC37A4), where glucose-6-phosphatase (G6PC) hydrolyzes it to glucose and inorganic phosphate (Pi). Glucose is recycled or exported. Associated disorders include GSD1b (G6PT/G6PC1 mutations), G6PC2 deficiency (pancreas-specific), and ubiquitously expressed G6PC3 defects.

Glucose transport across the endoplasmic reticulum (ER) membrane is governed by glucose transporter proteins (GLUTs) and the glucose-6-phosphate transporter (G6PT). ER membrane-integrated GLUTs mediate unidirectional glucose transport along concentration gradients, while G6PT catalyzes G6P/glucose antiport to sustain the electrochemical gradient. This process is integral to glucose metabolism, ensuring the cell’s supply of glucose for energy production and biosynthetic processes (Fig. 2).

### Inhibition of key signaling pathways in EMP of CRC

The metabolic reprogramming in CRC is significantly influenced by the activity of various signaling pathways that regulate the EMP pathway.

AKT, also known as protein kinase B, a PI3K-dependent serine/threonine kinase, critically regulates energy metabolism in CRC cells. AKT enhances glucose uptake by increasing the expression of Glut1, thereby promoting EMP [59]. Inhibition of the TGF-β/PI3K-AKT-mTOR signaling pathway in CRC cells downregulates Glut1, significantly reducing glucose uptake and blocking EMP. Furthermore, AKT activates HIF-1α to transcriptionally upregulate glycolytic effectors including HKII, PDHK1, and LDHA [12]. The natural compound wogonin reverses acquired drug resistance by dual PI3K/AKT pathway inhibition and HIF-1α downregulation, thereby silencing metabolic genes and impairing hypoxic adaptation in CRC cells. AKT also directly phosphorylates and activates the rate-limiting enzyme PK, enhancing EMP [60]. Collectively, AKT pathway targeting disrupts CRC bioenergetics, inducing metabolic starvation as a therapeutic strategy.

AMP-activated protein kinase (AMPK) is a highly conserved heterotrimer composed of catalytic subunits α (α1/α2) and regulatory subunits β (β1/β2) and γ (γ1/γ2/γ3), functions as a central cellular energy sensor coordinating ATP production-consumption balance. Activated AMPK can phosphorylate downstream substrates, upregulate Glut1, and increase glucose uptake, promoting EMP in CRC cells, synergistically modulated through its mammalian target of rapamycin (mTOR) pathway interactions [61]. Beyond canonical AMP-triggered activation, glucose deprivation also activates AMPK to shift metabolism toward catabolism. In CRC cells, the inhibition of AMPKα/p53 signaling pathway can promote metabolic transformation, leading to the transcriptional expression of phosphoglycerate mutase (PGM) and an increase in EMP. The degree of this defect is positively correlated with the efficiency of EMP and the malignancy of CRC [62]. AMPK also regulates the expression and activity of Glut and other EMP rate-limiting enzymes such as PFK, affecting EMP and glucose uptake in CRC cells. These findings elucidate the AMPK-EMP crosstalk in CRC, demonstrating how CRC cells modulate energy metabolism via AMPK-regulated glycolytic reprogramming.

The mTOR is a highly conserved serine/threonine protein kinase, functions through two distinct complexes: mTOR complex 1 (mTORC1; comprising mTOR, Raptor, AKT1, and mLST8/GβL) governing nutrient sensing and energy metabolism and mTOR complex 2 (mTORC2; consisting of mTOR, mLST8/GβL, Rictor, and Sin1) regulating cytoskeletal dynamics and survival. Approximately 40% of CRC cases exhibit dysregulated mTOR signaling pathway components [63]. PI3K/AKT-activated mTORC1 enhances glucose uptake via Glut1 upregulation. mTORC1 can also target the regulation of HIF-1α-mediated EMP to modulate the energy metabolism of HT-29 cells. Therefore, effectively inhibiting the mTOR/HIF-1α axis is one of the important means of anti-CRC therapy. mTORC2 influences CRC metabolism through AKT hyperactivation and c-Myc upregulation, linking oncogenic signaling to metabolic adaptation [64]. Targeting the mTOR signaling axis represents a pivotal therapeutic strategy in CRC, as its inhibition disrupts tumor bioenergetics metabolism, suppresses glycolytic flux (EMP), and ultimately constrains neoplastic proliferation and metastasis.

### Inhibition of key transcription factors in EMP of CRC

As master epigenetic modulators, transcription factors govern CRC’s metabolic rewiring through direct EMP pathway modulation.

**Fig. 3:**
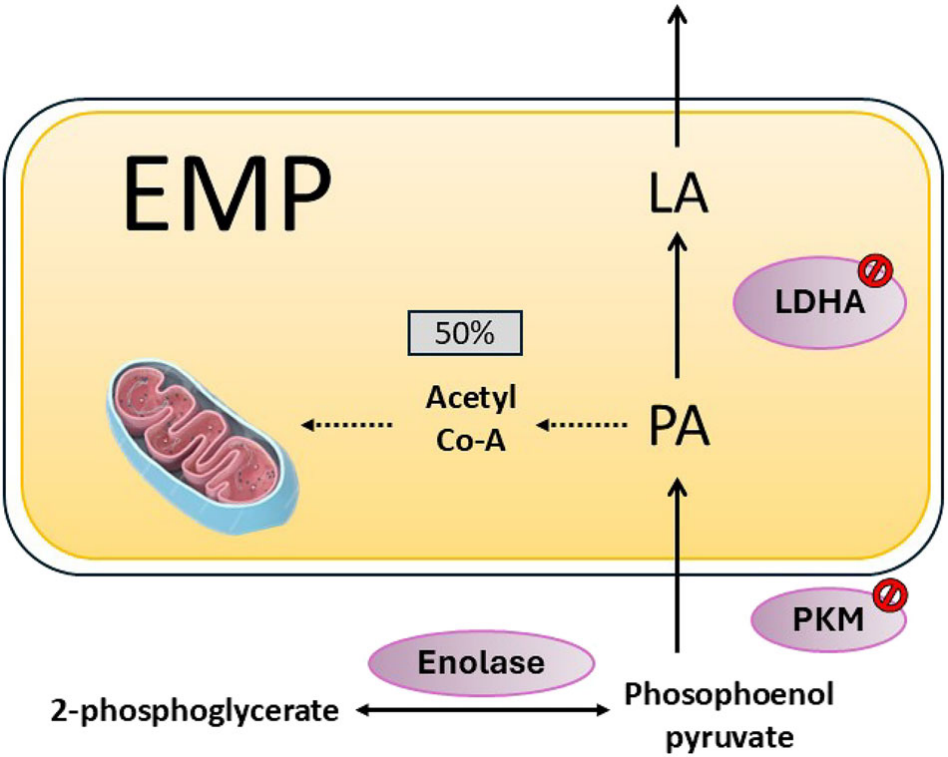
Inhibition targets in the EMP pathway of CRC cells. Converts glucose to pyruvate via enzymatic steps (e.g., enolase produces phosphoenolpyruvate). Inhibiting LDHA reduces lactate formation; PKM inhibition blocks phosphoenolpyruvate-to-pyruvate conversion. Half the flux diverts to mitochondrial acetyl-CoA for the TCA cycle. This balances energy production and metabolic intermediates under cellular demands. Solid lines: direct conversion of metabolites; Dashed lines: multi-step transformation of metabolites.

The tumor suppressor p53 regulates cell cycle progression and apoptosis while orchestrating metabolic reprogramming in malignancies by balancing OXPHOS and glycolysis. TIGAR, a p53-induced gene, has been identified as an effective inhibitor of EMP, redirecting energy utilization towards the PPP. TIGAR reduces the cellular levels of fructose-2,6-bisphosphate (FRU-2,6-P2), which drives metabolism towards EMP and inhibits gluconeogenesis, thus suppressing EMP and elevating NADPH levels [65]. However, p53 mutations/deletions in CRC cells enhance glycolytic dependency, driving cell cycle progression (G2/M → G1 transition) and energy production predominantly through glycolysis [66]. Additionally, PGM, another p53 target gene, catalyzes EMP when p53 is downregulated, thereby promoting EMP [67] (Fig. 3).

p53’s metabolic functions are multifaceted, as it suppresses anabolic pathways (de novo fatty acid/nucleotide synthesis) and activates catabolic processes (OXPHOS, lipolysis, β-oxidation), thereby restraining cancer cell proliferation through metabolic control [68]. However, the role of p53 in metabolic regulation is complex and can exhibit bidirectional activities, reflecting the intricate nature of p53’s functions. For instance, p53 can demonstrate antioxidant activity at physiological ROS levels to preserve cellular integrity but paradoxically amplifies ROS under oxidative stress to eliminate irreparably damaged cells while protecting neighboring tissues [69].

In CRC, the p53-induced gene TIGAR (TP53-induced glycolysis and apoptosis regulator) has been identified as an effective inhibitor of the EMP, redirecting energy utilization towards the PPP [70]. TIGAR suppresses EMP and elevates NADPH levels through dual mechanisms: reducing cellular levels of fructose-2,6-bisphosphate (FRU-2,6-P2), a key driver of metabolism towards the EMP, and inhibiting gluconeogenesis. This regulatory axis is clinically significant, as in CRC cells where p53 is silenced, the EMP becomes more active, promoting cell transition from G2~M to G1 phase and relying primarily on the EMP for energy supply [71]. Additionally, PGM, another p53 target gene, amplifies EMP activity under p53 loss, thereby promoting the EMP.

The coordinated regulation of EMP by the p53-TIGAR-PGM axis positions these transcriptional regulators and their metabolic effectors as actionable therapeutic targets in CRC. Future research directions could focus on understanding the precise mechanisms by which p53 and its associated proteins regulate the EMP in CRC, translating these mechanistic insights into dependency-targeting therapeutics that offer alternative treatment paradigms.

c-Myc overexpressed in ~70% of CRC patients [72], orchestrates glutamine, serine, and glycogen metabolism. Its phosphorylation sustains tumor proliferation under hypoxia, while pharmacological inhibition via acetoacetate suppresses downstream glycolytic flux, crippling CRC energy production [73]. c-Myc also regulates several key EMP enzymes, including Glut1, PKM2, and LDHA [74], increasing tumor cell uptake of glucose and production of pyruvate. Furthermore, c-Myc is implicated in the regulation of tumor cell energy metabolism through other metabolic pathways. Hypoxia-inducible NDRG2 disrupts this axis by downregulating ASCT2 glutamine transporters and glutaminase I via β-catenin-mediated c-Myc suppression, thereby blocking glycolytic adaptation. Collectively, targeting c-Myc and its downstream metabolic effects could offer a promising approach to disrupt the metabolic dependencies of CRC cells, potentially leading to novel therapeutic interventions.

Hypoxia-inducible factor 1α (HIF-1α) is a transcription factor induced under hypoxic conditions, enabling cells to adapt to low oxygen or anoxic conditions. CRC’s dysregulated proliferation fosters hypoxic TME driving HIF-1α overexpression [75]. HIF-1α induces the expression of EMP-related genes and enzymes to provide ample energy, mediated in CRC by HIF-1α-regulated Glut1 and Glut4. HIF-1α further interacts with Smad3 via microRNA-mediated regulation to upregulate the transcriptional levels of HKII and MCT4, intensifying glycolytic metabolism. HIF-1α inhibition (e.g., IDF-11774) in HCT116 cells suppresses LA and ATP production while elevating AMP/ATP ratios, confirming its glycolytic regulatory role [76]. These findings establish HIF-1α as a druggable nexus in CRC’s energy rewiring, offering rational targeting opportunities from an energy metabolism perspective.

## Metabolic reprogramming and immunotherapy in CRC

CRC pathogenesis involves oncogene-driven metabolic rewiring, a process that adaptively reconstitutes metabolic networks to sustain tumor proliferation and viability. As the metabolic cornerstone of cancer adaptation, Warburg-driven aerobic glycolysis propagates tumor expansion and concomitantly engineers immunosuppressive TME architectures. This oncometabolite impairs cytotoxic T-cells function while CRC cells’ preference for glycolysis over OXPHOS further reinforces immune evasion mechanisms, critically shaping therapeutic responses to immunotherapy.

Immune checkpoint inhibitors (ICIs; e.g., anti-PD-1/PD-L1 antibodies), demonstrate clinical efficacy in CRC, particularly microsatellite instability-high (MSI-H) subtypes. However, the response to ICIs is heterogeneous, and biomarkers like PD-L1 expression levels have been correlated with treatment outcomes. CRC immunogenicity is metabolically regulated, for example, O-GlcNAcylated enolase 1 (ENO1) coordinates glycolytic flux and immune checkpoint expression to facilitate immune evasion. Therapeutic targeting of glycolytic enzymes (HK, phosphofructokinase, and PK) can disrupt the metabolic fitness of CRC cells, making them more susceptible to immune-mediated killing. Additionally, these metabolic interventions can potentially reverse the immunosuppressive phenotype of the TME, enhancing the efficacy of ICIs [77, 78].

The metabolic heterogeneity of CRC dictates immunotherapy responses. MSI-H CRC tumors, which have a high mutational burden, are more likely to respond to ICIs due to increased antigen presentation. In contrast, microsatellite-stable CRC tumors, representing most cases, are less immunogenic and pose a greater challenge for immunotherapy. Deciphering subtype-specific metabolic signatures is crucial for developing personalized immunotherapeutic strategies.

Despite the potential of metabolic targeting in CRC, several clinical challenges remain due to tumor heterogeneity and microenvironment complexity, necessitating multidimensional strategies combining metabolic inhibitors with ICIs. Clinical trials are needed to identify biomarkers that predict response to metabolic interventions and to determine the optimal combination therapies that can overcome resistance mechanisms. Future research should focus on understanding the metabolic dependencies of CRC subtypes and developing combinatorial therapies that target both metabolic and immunological pathways. Metabolic reprogramming in CRC is intricately linked to immune evasion and response to immunotherapy. Emerging evidence reinforces the metabolic-immune interplay in CRC progression, where metabolic rewiring offers synergistic potential to potentiate immunotherapy efficacy, particularly in ICI-refractory subtypes. As our understanding of the metabolic-immunological axis in CRC advances, so too will our ability to develop personalized treatment strategies that harness the immune system to combat this disease [79, 80].

## Conclusion

Metabolic reprogramming critically drives CRC pathogenesis. Current research indicates that dysregulated glucose, lipids, and glutamine fuel CRC cell proliferation, invasion, and migration through key metabolic enzymes. This mechanistic understanding positions metabolic pathway targeting as a strategic focus in CRC therapeutic development.

Enzymes governing glycolytic flux in CRC cells exhibit crosstalk with TME dynamics, while EMP inhibitors demonstrate potential to mitigate treatment resistance to radiotherapy and chemotherapy. These findings position multi-targeted EMP enzyme inhibition as a promising therapeutic strategy. However, monotherapeutic approaches risk incomplete pathway suppression and multidrug resistance induction, necessitating development of combinatorial regimens targeting multiple EMP nodes to achieve durable therapeutic efficacy.

While EMP remains the predominant metabolic pathway for energy metabolism in CRC cells, most tumor cells’ mitochondrial function persists, enabling partial independence from glycolytic metabolism. OXPHOS-mediated metabolic compensation in CRC cells may compromise EMP-targeted drugs. Concurrent targeting of complementary pathways, such as the tricarboxylic acid cycle, PPP, and glutamine metabolism, represents a critical research frontier for comprehensive CRC metabolic intervention.

EMP inhibitors carry on-target toxicities for normal tissues dependent on glycolytic metabolism (e.g., brain, retina, and testes), necessitating tumor-selective therapeutic strategies. Critical research priorities include elucidating mechanisms for CRC-specific glycolytic blockade that spares normal cell viability, coupled with the development of multi-target regimens co-inhibiting EMP and complementary pathways to optimize therapeutic indices.

In conclusion, while substantial advances have elucidated metabolic reprogramming’s role in CRC, therapeutic development demands precision strategies addressing pathway complexity and TME crosstalk. Advancing CRC therapeutics requires systematic identification of context-specific metabolic targets and synergistic combination regimens that maximize antitumor efficacy while preserving normal tissue homeostasis.

## References

[1] Morgan E, Arnold M, Gini A, Lorenzoni V, Cabasag CJ, Laversanne M, et al. Global burden of colorectal cancer in 2020 and 2040: incidence and mortality estimates from GLOBOCAN. Gut. 2023;72:338–44. 36604116 10.1136/gutjnl-2022-327736

[2] Pinheiro M, Moreira DN, Ghidini M. Colon and rectal cancer: an emergent public health problem. World J Gastroenterol. 2024;30:644–51. 38515957 10.3748/wjg.v30.i7.644 PMC10950624

[3] Zhong X, He X, Wang Y, Hu Z, Huang H, Zhao S, et al. Warburg effect in colorectal cancer: the emerging roles in tumor microenvironment and therapeutic implications. J Hematol Oncol. 2022;15:160. 36319992 10.1186/s13045-022-01358-5 PMC9628128

[4] Nicolini A, Ferrari P. Involvement of tumor immune microenvironment metabolic reprogramming in colorectal cancer progression, immune escape, and response to immunotherapy. Front Immunol. 2024;15:1353787. 39119332 10.3389/fimmu.2024.1353787 PMC11306065

[5] Offermans K, Jenniskens JCA, Simons C, Samarska I, Fazzi GE, van der Meer JRM, et al. Association between mutational subgroups, Warburg-subtypes, and survival in patients with colorectal cancer. Cancer Med. 2023;12:1137–56. 35785488 10.1002/cam4.4968 PMC9883416

[6] Lu J, Kornmann M, Traub B. Role of epithelial to mesenchymal transition in colorectal cancer. Int J Mol Sci. 2023;24:1–21. 10.3390/ijms241914815 PMC10573312 37834263

[7] Ungefroren H, Thurling I, Farber B, Kowalke T, Fischer T, De Assis LVM, et al. The quasimesenchymal pancreatic ductal epithelial cell line PANC-1-A useful model to study clonal heterogeneity and EMT subtype shifting. Cancers. 2022;14:1–22. 10.3390/cancers14092057 PMC9101310 35565186

[8] Dong S, Liang S, Cheng Z, Zhang X, Luo L, Li L, et al. ROS/PI3K/Akt and Wnt/beta-catenin signalings activate HIF-1alpha-induced metabolic reprogramming to impart 5-fluorouracil resistance in colorectal cancer. J Exp Clin Cancer Res. 2022;41:15. 34998404 10.1186/s13046-021-02229-6 PMC8742403

[9] Sedlak JC, Yilmaz OH, Roper J. Metabolism and colorectal cancer. Ann Rev Pathol. 2023;18:467–92. 36323004 10.1146/annurev-pathmechdis-031521-041113 PMC9877174

[10] Jeong KY. Inhibiting focal adhesion kinase: a potential target for enhancing therapeutic efficacy in colorectal cancer therapy. World J Gastrointest Oncol. 2018;10:290–2. 30364839 10.4251/wjgo.v10.i10.290 PMC6198301

[11] Nalli M, Puxeddu M, La Regina G, Gianni S, Silvestri R. Emerging therapeutic agents for colorectal cancer. Molecules. 2021;26:1–26. 10.3390/molecules26247463 PMC8707340 34946546

[12] Liu S, Zhang X, Wang W, Li X, Sun X, Zhao Y, et al. Metabolic reprogramming and therapeutic resistance in primary and metastatic breast cancer. Mol Cancer. 2024;23:1–57. 39574178 10.1186/s12943-024-02165-x PMC11580516

[13] Qin R, Fan X, Huang Y, Chen S, Ding R, Yao Y, et al. Role of glucose metabolic reprogramming in colorectal cancer progression and drug resistance. Transl Oncol. 2024;50:102156. 39405607 10.1016/j.tranon.2024.102156 PMC11736406

[14] Li TY, Sun Y, Liang Y, Liu Q, Shi Y, Zhang C-S, et al. ULK1/2 constitute a bifurcate node controlling glucose metabolic fluxes in addition to autophagy. Mol Cell. 2016;62:359–70. 27153534 10.1016/j.molcel.2016.04.009

[15] Fang Y, Yan C, Zhao Q, Xu J, Liu Z, Gao J, et al. The roles of microbial products in the development of colorectal cancer: a review. Bioengineered. 2021;12:720–35. 33618627 10.1080/21655979.2021.1889109 PMC8806273

[16] Liberti MV, Locasale JW. The Warburg effect: how does it benefit cancer cells? Trends Biochem Sci. 2016;41:211–8. 26778478 10.1016/j.tibs.2015.12.001 PMC4783224

[17] Guan Y, Yao W, Yu H, Feng Y, Zhao Y, Zhan X, et al. Chronic stress promotes colorectal cancer progression by enhancing glycolysis through beta2-AR/CREB1 signal pathway. Int J Biol Sci. 2023;19:2006–19. 37151872 10.7150/ijbs.79583 PMC10158030

[18] Zhou D, Yao Y, Zong L, Zhou G, Feng M, Chen J, et al. TBK1 facilitates GLUT1-dependent glucose consumption by suppressing mTORC1 signaling in colorectal cancer progression. Int J Biol Sci. 2022;18:3374–89. 35637944 10.7150/ijbs.70742 PMC9134896

[19] Wang J, Zhu M, Zhu J, Li J, Zhu X, Wang K, et al. HES1 promotes aerobic glycolysis and cancer progression of colorectal cancer via IGF2BP2-mediated GLUT1 m6A modification. Cell Death Discov. 2023;9:411. 37957183 10.1038/s41420-023-01707-4 PMC10643658

[20] Liu Y, Zhang Q, Xing B, Luo N, Gao R, Yu K, et al. Immune phenotypic linkage between colorectal cancer and liver metastasis. Cancer Cell. 2022;40:424–37 e5. 35303421 10.1016/j.ccell.2022.02.013

[21] Zheng Z, Wieder T, Mauerer B, Schafer L, Kesselring R, Braumuller H. T cells in colorectal cancer: unravelling the function of different T cell subsets in the tumor microenvironment. Int J Mol Sci. 2023;24:1–35. 10.3390/ijms241411673 PMC10380781 37511431

[22] Lv Y, Tang W, Xu Y, Chang W, Zhang Z, Lin Q, et al. Apolipoprotein L3 enhances CD8+ T cell antitumor immunity of colorectal cancer by promoting LDHA-mediated ferroptosis. Int J Biol Sci. 2023;19:1284–98. 36923931 10.7150/ijbs.74985 PMC10008698

[23] Zhang K, Zhang T, Yang Y, Tu W, Huang H, Wang Y, et al. N(6)-methyladenosine-mediated LDHA induction potentiates chemoresistance of colorectal cancer cells through metabolic reprogramming. Theranostics. 2022;12:4802–17. 35832094 10.7150/thno.73746 PMC9254245

[24] Hirose Y, Taniguchi K. Intratumoral metabolic heterogeneity of colorectal cancer. Am J Physiol Cell Physiol. 2023;325:C1073–c84. 37661922 10.1152/ajpcell.00139.2021

[25] Li C, Chen T, Liu J, Wang Y, Zhang C, Guo L, et al. FGF19-induced inflammatory CAF promoted neutrophil extracellular trap formation in the liver metastasis of colorectal cancer. Adv Sci. 2023;10:e2302613. 10.1002/advs.202302613 PMC10460854 37345586

[26] Kamali Zonouzi S, Pezeshki PS, Razi S, Rezaei N. Cancer-associated fibroblasts in colorectal cancer. Clin Transl Oncol. 2022;24:757–69. 34839457 10.1007/s12094-021-02734-2

[27] Nenkov M, Ma Y, Gaßler N, Chen Y. Metabolic reprogramming of colorectal cancer cells and the microenvironment: implication for therapy. Int J Mol Sci. 2021;22:6262. 34200820 10.3390/ijms22126262 PMC8230539

[28] Huang A, Sun Z, Hong H, Yang Y, Chen J, Gao Z, et al. Novel hypoxia- and lactate metabolism-related molecular subtyping and prognostic signature for colorectal cancer. J Transl Med. 2024;22:587. 38902737 10.1186/s12967-024-05391-5 PMC11191174

[29] Yue Y, She X, Ding W, Chen S, Xiao Q, Pan B, et al. A novel senescence-based prognostic model unveils tumor interactions and drug resistance in colorectal cancer. Int Immunopharmacol. 2024;134:112197. 38733826 10.1016/j.intimp.2024.112197

[30] Zhu W, Yang S, Meng D, Wang Q, Ji J. Targeting NADPH oxidase and integrin alpha5beta1 to inhibit neutrophil extracellular traps-mediated metastasis in colorectal cancer. Int J Mol Sci. 2023;24:1–27. 10.3390/ijms242116001 PMC10650826 37958984

[31] Patra KC, Hay N. The pentose phosphate pathway and cancer. Trends Biochem Sci. 2014;39:347–54. 25037503 10.1016/j.tibs.2014.06.005 PMC4329227

[32] TeSlaa T, Ralser M, Fan J, Rabinowitz JD. The pentose phosphate pathway in health and disease. Nat Metab. 2023;5:1275–89. 37612403 10.1038/s42255-023-00863-2 PMC11251397

[33] Song J, Sun H, Zhang S, Shan C. The multiple roles of glucose-6-phosphate dehydrogenase in tumorigenesis and cancer chemoresistance. Life. 2022;12:1–19. 10.3390/life12020271 PMC8875868 35207558

[34] Jin L, Zhou Y. Crucial role of the pentose phosphate pathway in malignant tumors. Oncol Lett. 2019;17:4213–21. 30944616 10.3892/ol.2019.10112 PMC6444344

[35] Yoon SJ, Combs JA, Falzone A, Prieto-Farigua N, Caldwell S, Ackerman HD, et al. Comprehensive metabolic tracing reveals the origin and catabolism of cysteine in mammalian tissues and tumors. Cancer Res. 2023;83:1426–42. 36862034 10.1158/0008-5472.CAN-22-3000 PMC10152234

[36] Zhang F, Wu Z, Yu B, Ning Z, Lu Z, Li L, et al. ATP13A2 activates the pentose phosphate pathway to promote colorectal cancer growth though TFEB-PGD axis. Clin Transl Med. 2023;13:e1272. 37243374 10.1002/ctm2.1272 PMC10220388

[37] Li K, Liu W, Yu H, Chen J, Tang W, Wang J, et al. 68Ga-FAPI PET imaging monitors response to combined TGF-betaR inhibition and immunotherapy in metastatic colorectal cancer. J Clin Investig. 2024;134:1–16. 10.1172/JCI170490 PMC10866654 38175716

[38] Lund M, Bjerre TA, Gronbaek H, Mortensen FV, Andersen PK. CEUS compared with CECT, MRI, and FDG-PET/CT for diagnosing CRC liver metastases: a diagnostic test accuracy systematic review and meta-analysis. Expert Rev Gastroenterol Hepatol. 2024;18:541–9. 39315472 10.1080/17474124.2024.2407973

[39] Sobhani I, Itti E, Luciani A, Baumgaertner I, Layese R, Andre T, et al. Colorectal cancer (CRC) monitoring by 6-monthly 18FDG-PET/CT: an open-label multicentre randomised trial. Ann Oncol. 2018;29:931–7. 29365058 10.1093/annonc/mdy031 PMC5913635

[40] Agarwal A, Marcus C, Xiao J, Nene P, Kachnic LA, Subramaniam RM. FDG PET/CT in the management of colorectal and anal cancers. Am J Roentgenol. 2014;203:1109–19. 25341152 10.2214/AJR.13.12256

[41] Shi Y, Wang M, Zhang J, Xiang Z, Li C, Zhang J, et al. Tailoring the clinical management of colorectal cancer by (18)F-FDG PET/CT. Front Oncol. 2022;12:1062704. 36620584 10.3389/fonc.2022.1062704 PMC9814158

[42] Komek H, Can C, Kaplan I, Gundogan C, Kepenek F, Karaoglan H, et al. Comparison of [(68) Ga]Ga-DOTA-FAPI-04 PET/CT and [(18)F]FDG PET/CT in colorectal cancer. Eur J Nucl Med Mol Imaging. 2022;49:3898–909. 35578038 10.1007/s00259-022-05839-0

[43] Guiot J, Vaidyanathan A, Deprez L, Zerka F, Danthine D, Frix AN, et al. A review in radiomics: making personalized medicine a reality via routine imaging. Med Res Rev. 2022;42:426–40. 34309893 10.1002/med.21846

[44] Gao R, Wu C, Zhu Y, Kong C, Zhu Y, Gao Y,et al. Integrated analysis of colorectal cancer reveals cross-cohort gut microbial signatures and associated serum metabolites. Gastroenterology. 2022;163:1024–37 e9. 35788345 10.1053/j.gastro.2022.06.069

[45] Yun Z, Guo Z, Li X, Shen Y, Nan M, Dong Q, et al. Genetically predicted 486 blood metabolites in relation to risk of colorectal cancer: a Mendelian randomization study. Cancer Med. 2023;12:13784–99. 37132247 10.1002/cam4.6022 PMC10315807

[46] Stefani C, Miricescu D, Stanescu S, II, Nica RI, Greabu M, Totan AR, et al. Growth factors, PI3K/AKT/mTOR and MAPK signaling pathways in colorectal cancer pathogenesis: where are we now? Int J Mol Sci. 2021;221:1–24. 10.3390/ijms221910260 PMC8508474 34638601

[47] Yang Y, Liu Q, Wang M, Li L, Yu Y, Pan M, et al. Genetically programmable cell membrane-camouflaged nanoparticles for targeted combination therapy of colorectal cancer. Signal Transduct Target Ther. 2024;9:158. 38862461 10.1038/s41392-024-01859-4 PMC11167040

[48] Zhao L, Yu N, Zhai Y, Yang Y, Wang Y, Yang Y, et al. The ubiquitin-like protein UBTD1 promotes colorectal cancer progression by stabilizing c-Myc to upregulate glycolysis. Cell Death Dis. 2024;15:502. 39003255 10.1038/s41419-024-06890-5 PMC11246417

[49] Huang Y, Xiong C, Wang C, Deng J, Zuo Z, Wu H, et al. p53-responsive CMBL reprograms glucose metabolism and suppresses cancer development by destabilizing phosphofructokinase PFKP. Cell Rep. 2023;42:113426. 37967006 10.1016/j.celrep.2023.113426

[50] Zhao X, Qi X, Lian W, Tong X, Wang H, Su L, et al. Trichomicin suppresses colorectal cancer via comprehensive regulation of IL-6 and TNFα in tumor cells, TAMs, and CAFs. Front Pharmacol. 2020;11:386. 32317968 10.3389/fphar.2020.00386 PMC7146085

[51] Wang M, Flaswinkel H, Joshi A, Napoli M, Masgrau-Alsina S, Kamper JM, et al. Phosphorylation of PFKL regulates metabolic reprogramming in macrophages following pattern recognition receptor activation. Nat Commun. 2024;15:6438. 39085210 10.1038/s41467-024-50104-7 PMC11291651

[52] Wei W, Zhang ZY, Shi B, Cai Y, Zhang HS, Sun CL, et al. METTL16 promotes glycolytic metabolism reprogramming and colorectal cancer progression. J Exp Clin Cancer Res. 2023;42:151. 37340443 10.1186/s13046-023-02732-y PMC10280857

[53] Yin K, Lee J, Liu Z, Kim H, Martin DR, Wu D, et al. Mitophagy protein PINK1 suppresses colon tumor growth by metabolic reprogramming via p53 activation and reducing acetyl-CoA production. Cell Death Differ. 2021;28:2421–35. 33723373 10.1038/s41418-021-00760-9 PMC8329176

[54] Ginés A, Bystrup S, Ruiz de Porras V, Guardia C, Musulén E, Martínez-Cardús A, et al. PKM2 subcellular localization is involved in oxaliplatin resistance acquisition in HT29 human colorectal cancer cell lines. PLoS ONE. 2015;10:e0123830. 25955657 10.1371/journal.pone.0123830 PMC4425499

[55] Guan X, Liu R, Wang B, Xiong R, Cui L, Liao Y, et al. Inhibition of HDAC2 sensitises antitumour therapy by promoting NLRP3/GSDMD-mediated pyroptosis in colorectal cancer. Clin Transl Med. 2024;14:e1692. 38804602 10.1002/ctm2.1692 PMC11131357

[56] Macharia JM, Kaposztas Z, Varjas T, Budan F, Zand A, Bodnar I, et al. Targeted lactate dehydrogenase genes silencing in probiotic lactic acid bacteria: a possible paradigm shift in colorectal cancer treatment? Biomed Pharmacother. 2023;160:114371. 36758316 10.1016/j.biopha.2023.114371

[57] Kasprzak A. Insulin-like growth factor 1 (IGF-1) signaling in glucose metabolism in colorectal cancer. Int J Mol Sci. 2021;22:1–41. 10.3390/ijms22126434 PMC8234711 34208601

[58] Zhao G, Yuan H, Li Q, Zhang J, Guo Y, Feng T, et al. DDX39B drives colorectal cancer progression by promoting the stability and nuclear translocation of PKM2. Signal Transduct Target Ther. 2022;7:275. 35973989 10.1038/s41392-022-01096-7 PMC9381590

[59] Duan S, Huang W, Liu X, Liu X, Chen N, Xu Q, et al. IMPDH2 promotes colorectal cancer progression through activation of the PI3K/AKT/mTOR and PI3K/AKT/FOXO1 signaling pathways. J Exp Clin Cancer Res. 2018;37:304. 30518405 10.1186/s13046-018-0980-3 PMC6282329

[60] Yao PA, Wu Y, Zhao K, Li Y, Cao J, Xing C. The feedback loop of ANKHD1/lncRNA MALAT1/YAP1 strengthens the radioresistance of CRC by activating YAP1/AKT signaling. Cell Death Dis. 2022;13:103. 35110552 10.1038/s41419-022-04554-w PMC8810793

[61] Chan S, Wang X, Wang Z, Du Y, Zuo X, Chen J, et al. CTSG Suppresses colorectal cancer progression through negative regulation of Akt/mTOR/Bcl2 signaling pathway. Int J Biol Sci. 2023;19:2220–33. 37151875 10.7150/ijbs.82000 PMC10158020

[62] Maharati A, Moghbeli M. PI3K/AKT signaling pathway as a critical regulator of epithelial-mesenchymal transition in colorectal tumor cells. Cell Commun Signal. 2023;21:201. 37580737 10.1186/s12964-023-01225-x PMC10424373

[63] Xu T, Li X, Zhao W, Wang X, Jin L, Feng Z, et al. SF3B3-regulated mTOR alternative splicing promotes colorectal cancer progression and metastasis. J Exp Clin Cancer Res. 2024;43:126. 38671459 10.1186/s13046-024-03053-4 PMC11047005

[64] Yan H, Talty R, Johnson CH. Targeting ferroptosis to treat colorectal cancer. Trends Cell Biol. 2023;33:185–8. 36473802 10.1016/j.tcb.2022.11.003

[65] Tang J, Chen L, Qin ZH, Sheng R. Structure, regulation, and biological functions of TIGAR and its role in diseases. Acta Pharmacol Sin. 2021;42:1547–55. 33510458 10.1038/s41401-020-00588-y PMC8463536

[66] Li XL, Zhou J, Chen ZR, Chng WJ. P53 mutations in colorectal cancer - molecular pathogenesis and pharmacological reactivation. World J Gastroenterol. 2015;21:84–93. 25574081 10.3748/wjg.v21.i1.84 PMC4284363

[67] Liu C, Rokavec M, Huang Z, Hermeking H. Curcumin activates a ROS/KEAP1/NRF2/miR-34a/b/c cascade to suppress colorectal cancer metastasis. Cell Death Differ. 2023;30:1771–85. 37210578 10.1038/s41418-023-01178-1 PMC10307888

[68] Huang D, Sun W, Zhou Y, Li P, Chen F, Chen H, et al. Mutations of key driver genes in colorectal cancer progression and metastasis. Cancer Metastasis Rev. 2018;37:173–87. 29322354 10.1007/s10555-017-9726-5

[69] Malki A, ElRuz RA, Gupta I, Allouch A, Vranic S, Al Moustafa AE. Molecular mechanisms of colon cancer progression and metastasis: recent insights and advancements. Int J Mol Sci. 2020;22:1–23. 10.3390/ijms22010130 PMC7794761 33374459

[70] Liu MY, Li HM, Wang XY, Xia R, Li X, Ma YJ, et al. TIGAR drives colorectal cancer ferroptosis resistance through ROS/AMPK/SCD1 pathway. Free Radic Biol Med. 2022;182:219–31. 35271998 10.1016/j.freeradbiomed.2022.03.002

[71] Song C, Zhao L, Deng J, Wang L, Mao M, Peng S, et al. E2F8-induced GRPEL2 promoted colorectal cancer progression via targeting TIGAR. J Transl Med. 2025;23:466. 40269881 10.1186/s12967-025-06451-0 PMC12020167

[72] Fang Y, Shen ZY, Zhan YZ, Feng XC, Chen KL, Li YS, et al. CD36 inhibits beta-catenin/c-myc-mediated glycolysis through ubiquitination of GPC4 to repress colorectal tumorigenesis. Nat Commun. 2019;10:3981. 31484922 10.1038/s41467-019-11662-3 PMC6726635

[73] Jing Z, Liu Q, He X, Jia Z, Xu Z, Yang B, et al. NCAPD3 enhances Warburg effect through c-myc and E2F1 and promotes the occurrence and progression of colorectal cancer. J Exp Clin Cancer Res. 2022;41:198. 35689245 10.1186/s13046-022-02412-3 PMC9188166

[74] Di Y, Jing X, Hu K, Wen X, Ye L, Zhang X, et al. The c-MYC-WDR43 signalling axis promotes chemoresistance and tumour growth in colorectal cancer by inhibiting p53 activity. Drug Resist Updates. 2023;66:100909. 10.1016/j.drup.2022.100909 36525936

[75] Weng ML, Chen WK, Chen XY, Lu H, Sun ZR, Yu Q, et al. Fasting inhibits aerobic glycolysis and proliferation in colorectal cancer via the Fdft1-mediated AKT/mTOR/HIF1alpha pathway suppression. Nat Commun. 2020;11:1869. 32313017 10.1038/s41467-020-15795-8 PMC7170903

[76] Singhal R, Mitta SR, Das NK, Kerk SA, Sajjakulnukit P, Solanki S, et al. HIF-2alpha activation potentiates oxidative cell death in colorectal cancers by increasing cellular iron. J Clin Investig. 2021;131:1–15. 10.1172/JCI143691 PMC8203462 33914705

[77] Lin KX, Istl AC, Quan D, Skaro A, Tang E, Zheng X. PD-1 and PD-L1 inhibitors in cold colorectal cancer: challenges and strategies. Cancer Immunol Immunother. 2023;72:3875–93. 37831146 10.1007/s00262-023-03520-5 PMC10700246

[78] Jin Z, Sinicrope FA. Mismatch repair-deficient colorectal cancer: building on checkpoint blockade. J Clin Oncol. 2022;40:2735–50. 35649217 10.1200/JCO.21.02691 PMC9390830

[79] Li Q, Geng S, Luo H, Wang W, Mo Y-Q, Luo Q, et al. Signaling pathways involved in colorectal cancer: pathogenesis and targeted therapy. Signal Transduct Target Ther. 2024;9:266. 39370455 10.1038/s41392-024-01953-7 PMC11456611

[80] Ouladan S, Orouji E. Chimeric antigen receptor-T cells in colorectal cancer: pioneering new avenues in solid tumor immunotherapy. J Clin Oncol. 2025;43:994–1005. 10.1200/JCO-24-02081 PMC11895826 39805063

